# Medicine and Hygiene

**Published:** 1879-02

**Authors:** 


### Editorial.

### Medicine and Hygiene.

The history of medicine shows us that professional research has thus far been directed to the *cure* of disease—to the specific influence of drugs—to the chemical physiological and medicinal properties of the innumerable remedies already known and the discovery of new and hitherto unknown drugs or properties of drugs, while the prevention of disease, or hygiene is a new and, until quite recently, an unappreciated and mostly uncultivated field of labor.

The *cure* of disease by medicine has been skillfully and faithfully tried for two thousand years, and may be fairly and truthfully acknowledged to have *proved a failure.*

We may fairly claim wonderful achievements in the guidance and control of disease, in the relief and *care* of the sick, and in the correction of disordered function, but physicians have thus far failed to discover what can truthfully be denominated a *cure.* Mercury would probably come nearest to the distinction of any known drug, old and unfashionable as it is, but the wonderful effects of this drug upon syphilis cannot entitle it to the credit of being quite curative of this single disease. Iodine and mercury may be regarded as controlling in a most wonderful degree, but the medical profession of all denominations may as well confess that the scope of their influence does not include the *cure of disease.* But a wider and better field is now being or has been opened up to the profession of the present and more careful investigation of the future. We have only fairly commenced investigation of the *Hygienic* influences which determine health and disease, but here seems to offer a productive field for cultivation.

The medical colleges of the United States are about to issue a large installment of physicians and we are glad to be able to announce that there is an inviting field of labor opened up to them; a new mine but recently discovered, offering rich returns for faithful labor.

Pure air, suitable clothing, ample food and constant daily out-door life, would at present seem to be the essential of health and long life. Confined and unventilated over-crowded apartments, obstructed imperfect sewers, stagnant, decomposing accumulations of animal and vegetable matter, and uncleanness in all forms appear at the present point of knowledge to be important items in the causation of nearly all our diseases. We have written and talked about climate as influencing or determining the character, duration and termination of our diseases, but it requires no further proof that it is local hygienic conditions and not general climatic influences which determines in very great degree the differences between the healthfulness of our great eastern and western cities, and the climate of the territories or slopes of the Rocky Mountains.

We advise our patients to leave their city homes for the west or the south, forgetting for the time, at least, that our own state or city has naturally a climate as salubrious, invigorating and healthy as any place upon the earth where the sun shines. We are soon to graduate in Buffalo about fifty young men as physicians, and if they will all make hygiene a "*specialty*," and "not attend to anything else," there is ample employment for them all in this city, while in treating disease with drugs there is little room for even one.

This subject is too vast and important for complete canvas in the space allowed for editorial remark, and we have only attempted to partially outline it at present.

